# The Physical Fitness of the Nation

**Published:** 1918-02-16

**Authors:** 


### THE PHYSICAL FITNESS OF THE NATION.

Frequently, prior to the present war, and particularly in connection with the Boer War, loud lamentations were uttered over the physical deterioration of the peoples who inhabit the British Isles. We were not, so it was said, equal to our forefathers either in manly efficiency or physical courage. What standard was enforced in earlier days was not considered, and it seems at present to need some courage to assert that the British peoples are less effective in heroism and endurance and resolve than were the men who formerly fought to victory on all the continents. In truth our people have not come short, and no historian can say that they have failed to be worthy of their ancestry.

So much by way of comparison. But when we take the facts as they stand, and as revealed for example by the recruiting statistics, there are abundant reasons for saying that the physical fitness of large numbers of the nation leaves much to be desired, and this whether the judgment is based on military standards or on economic values. Whether in this respect our forefathers were better or worse than ourselves is a much less important question than that which asks whether we are as fit as we ought to be. Early in the present century an elaborate inquiry conducted under Government auspices sought to settle the first of these issues, and the answer was by no means a discouraging one. Though nothing practical has come from this inquiry we notice that the anthropologists are proposing another investigation, in which, presumably, various groups of the people are to be measured and labelled in regard to their physical qualities, and in this fashion the resources of science are to be placed at the disposal of the Government. We can profess no sympathy with the proposal. If the object is merely a sort of official measurement of the people, and the accumulation of official records, the game is hardly worth the candle, and we imagine that of Government inspectors and visitors most of us already have more than enough. On the other hand, if the suggestion is that such an inquiry may be made the basis for reforms which will improve the national health and fitness, we reply that a new investigation can tell us nothing that we do not know already. The picture in its broad outlines is plain enough. So long as people are crowded into mean streets and have a scanty supply of pure air and sunshine, so long there will be vicious habits and enfeebling diseases. To establish this conclusion it is not necessary to measure the facial angle of school children or the respiratory value of artisans' chests. The difficulty is to remove the causes which, it is notorious, are prejudicial to robust health and development, and this, on the economic plane, means the adjustment of the conditions of industrialism to the demands of hygienic law. If scientific societies can find a solution of this problem by all means let us hear it. But if they have nothing to offer except further measurements and more inquisitions we, to use a vulgar tag, are not taking any. Substantially we know the facts: what we want is statesmanship to deal with them.

